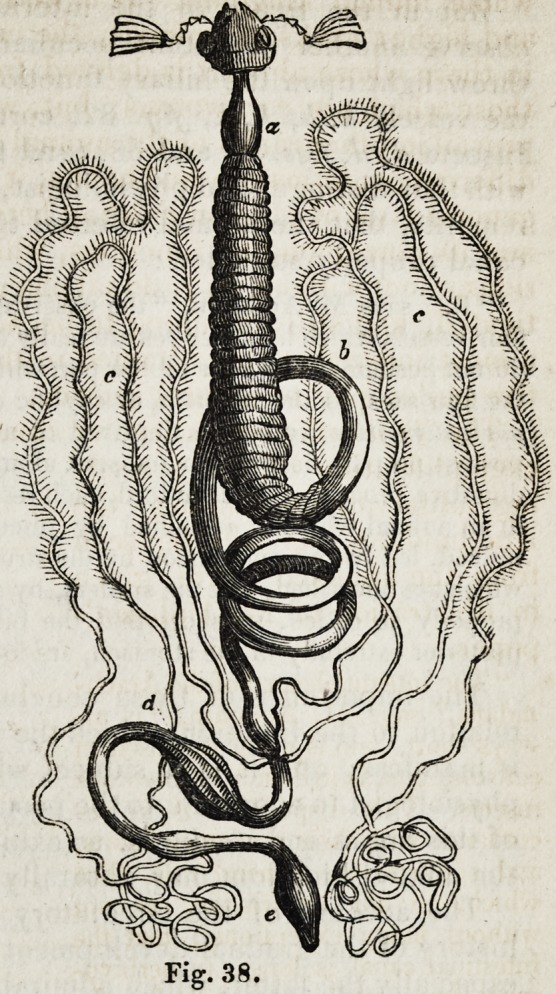# The Cyclopædia of Anatomy and Physiology

**Published:** 1836-04

**Authors:** 


Art. XVII.—*The Cyclopædia of Anatomy and Physiology.* Edited by Robert B. Todd, m.b. Parts I. to IV.—*London*, 1835. Royal 8vo. pp. 416.

From the editor's address prefixed to the first part of this work, we learn that "it is intended to embrace the whole of the sciences of Anatomy and Physiology, these terms being used in their largest sense, as far as regards the animal kingdom." In the filling up of this plan, as far as the Parts hitherto published enable us to form a judgment, the several essays would seem naturally to fall under four sections or departments, viz. Anatomy, Physiology, Comparative Anatomy, and Animal Chemistry. The anatomical section embraces papers upon general, descriptive, surgical, and morbid anatomy; and it is a feature of no inconsiderable merit in the original plan drawn out by Dr. Todd, that it admits of placing this most important branch of natural science in a light so varied, and at the same time so practically useful. Anatomy forms the groundwork, upon a competent knowledge of which many other branches of scientific enquiry must rest, and is the only sure foundation upon which all medical acquirements can be based. It is therefore a subject which will admit of our dwelling at some length upon the several articles relating to it in the important work before us; but various considerations compel us to deviate from our original intention on this point; and, among others, the desire to draw the attention of our readers to the admirable papers upon Comparative Anatomy. It would, however, be unjust entirely to pass over the articles upon Human Anatomy without giving the due meed of praise to their respective authors; and, if there is not much originality displayed in some of these essays, (which, indeed, from the nature of the subject, could scarcely have been expected,) still we are bound to admit that they are in general careful and judicious digests of the present state of our knowledge. The essay of the Editor upon the Abdomen, and that of Dr. Craigie upon the Adipose Tissue, are good examples of the manner in which the subjects belonging to regional and general anatomy are treated; and the Dublin School of Anatomy will not lose any of its well-earned reputation by the manner in which the gentlemen belonging to that School have treated the several articles allotted to them. In the physiological department also, which we must not altogether pass without notice, we find an article, of a very high standard of merit, upon Absorption, by Dr. Bostock, which affords an excellent compendium of what is known upon the subject, although perhaps scarcely allowing its due weight to the theory of imbibition. There is also a good account of Asphyxia by Professor Alison, the chief defect of which is a very unusual one—its brevity. Dr. Symonds's article, Age, is also excellent.

The study of comparative anatomy, and of the relations which it exhibits with anatomy (properly so called), is every day becoming more important; and the diligent investigation of the facts presented by this branch of scientific enquiry is calculated to throw light upon many of the dark places which yet remain to be explored in man's organization. The development of the intimate structure and functions of organs, which, in the more elaborate forms of the higher ranks of existence, appear complicated and obscure, is materially facilitated by contemplating the same organs and the same functions in beings of a more simple construction. Nature herself here becomes our handmaid in the unravelling of the intricate web, and thus displays many of her most secret processes to our view.

The papers upon Comparative Anatomy in these four parts of the Cyclopædia are, a General Sketch of the Animal Kingdom, by Dr. Grant; a brief notice of the *Articulata* and the *Acrita*, by Mr. Owen; the articles *Acalephæ*, *Annelida*, *Arachnida*, and *Amphibia*, by Dr. Coldstream, Dr. Milne Edwards, M. Audouin, and Mr. T. Bell; and an elaborate memoir upon the *Aves*, by Mr. Owen. These papers are all possessed of considerable merit. Dr. Grant's sketch affords a clear and concise introduction to the subject generally, an outline which is well filled up, as far as the work has hitherto advanced, by the authors of the articles devoted to the more particular consideration of the several classes. Dr. Audouin's account of the Arachnida is especially interesting, and it is to this that we propose upon the present occasion more particularly to direct our attention.

The Arachnidans are divided by Latreille, from certain peculiarities in their organs of respiration, into *Pulmonaries*, or those provided with pulmonary sacs, and *Trachearies*, or those breathing by tracheæ. The first of these divisions, the *Arachnida pulmonaria*, embraces two great families,—the *Araneidæ*, or spiders, admirably arranged into genera by M. Walcknäer; and the *Pedipalpi*, or scorpions. The second division, or Trachiaries, including the *Acaridæ*, or mites, and many other anomalous species, has been recently arranged by M. Duges; but has hitherto been very imperfectly studied, notwithstanding that the late researches upon one of this tribe, (the *Acarus scabiei*,) by M. Raspail and others, demonstrate its importance both in a scientific and in a practical point of view.

M. Audouin, after giving a brief summary of the natural characters distinctive of the class, and the classification now most generally adopted by zoologists,—viz. that of Latreille,—proceeds to describe, 1st, the external covering, or tegumentary system; 2d, the digestive system; 3d, the respiratory system; 4th, the circulating system; 5th, the nervous system; 6th, the organs of secretion; 7th, the generative system: concluding with a highly interesting account of the gradual development of the ova. We cannot attempt to follow M. Audouin into the numerous and important details which he has here brought before us, although the information to be derived from them is by no means confined to the class of beings which is the more especial subject of the essay. The observations upon the digestive apparatus, however, afford some peculiarly interesting facts, not only in respect to the Arachnidans themselves, but also in relation to the great question of assimilation throughout the whole animal kingdom. It is well known that, among the Mammalia and higher ranks of animals, the intestinal canal is considerably longer in those whose aliment is derived from the vegetable kingdom, than in those which are carnivorous; but, when the same peculiarity is found to prevail in other ranks of the animal kingdom, and among beings of very different conformation and habits, it affords us an insight into one of the general laws of relation existing between the organized animal structure and its corresponding functions, which, although not without its exceptions,—not without its residual phænomena requiring to be further investigated, and still remaining to be accounted for,—must be regarded as a most important step attained in the application of the rules of inductive philosophy to natural science.

The *Tegenaria domestica*, or common house-spider, affords a striking exemplification of the preceding observations among the Arachnidans, as will be seen from the annexed figure, given in illustration of M. Audouin's paper.

"The intestinal canal of the Arachnidans," says M. Audouin, "is always short, and is never disposed in convolutions, as in certain herbivorous insects. This disposition is in accordance with their predatory habits, and confirms the general rule, (but which, to our knowledge, is not without many exceptions,) that the intestinal canal is longer in herbivorous than carnivorous animals. In the spiders (Araneæ), and we may take the common species (*Tegenaria domestica*) as an example, the alimentary canal (*fig.* 82) communicates with the mouth between the maxillæ (*a a*,) by an œsophagus, rather short and of a delicate texture, (*b.*) This terminates in four sacs (*c*), which M. Treviranus calls stomach, but which, in our opinion, merit the name of gizzards: the digestive tube then continues, as a straight narrow canal (*d*), of moderate length, which dilates (*e*) and adheres, by its parietes, to a kind of epiploon filled with adipose granules (*f*). Posteriorly the dilated part becomes stronger in texture, insensibly contracts (*g*), then undergoes a second dilatation (*h*), before it opens into the rectum (*i*)."

The vessels, *k*, *k*, *k*, *k*, terminating near the rectum, are the biliary vessels of Treviranus, to which we shall presently revert.

Fig. 82.

Dr. Grant has pointed out the same peculiarity in the *Insecta*.

Fig. 37.

Fig. 38.

"In the adult state," he observes, "the masticating organs and the digestive apparatus vary much according to the kind of food in the different species, as is seen in comparing the alimentary canal of a carnivorous *cicindela campestris* (*fig.* 37,) with that of a phytophagous *melolontha vulgaris*, (*fig.* 38.) In the carnivorous insect (*fig.* 37,) the intestine passes nearly straight through the body, with few enlargements in its course, and the glandular organs have a simpler structure. The œsophagus passes down narrow from the head, and dilates into a wide glandular crop (*a*), which is succeeded by a minute gizzard, and this is followed by a chylific stomach (*b*, *c*), which is covered like the crop with minute glandular cryptæ or follicles. At the pyloric extremity of the chylific stomach, the liver, in form of simple biliary ducts, pours its secretion into that cavity by two orifices on each side (*d*). The short small intestine (e) opens into a wide colon (*f*), which terminates in the anus (*g*). In the vegetable-eating insect, (*fig.* 38), the alimentary canal is more lengthened, convoluted, and capacious, with more numerous dilations, and the glandular organs are more developed. The crop (*a*) of the *melolontha* is succeeded by a minute rudimentary gizzard, and to this succeeds a long and sacculated glandular or chylific stomach, which becomes narrow and convoluted below, and terminates in a small pyloric dilatation, which receives the four terminations of the biliary organs. The succeeding part of the intestine is also convoluted, and has three enlargements in its course to the anus (*e*). The liver (*cc*) is here of great magnitude, and has its secreting surface much extended by the development of innumerable minute cæca from its primary ducts."

The gasteropoda afford further illustrations of the same law, as may be seen by comparing the intestine of the carnivorous *Buccinum undatum* with that of the *Patella vulgata*, which feeds on marine plants.*

* See figures 41 and 42, and Dr. Grant's observations upon them, Part II. p. 113.

But in the figure of the internal anatomy of the house spider we observe another important peculiarity of conformation, which tends to throw light upon the biliary function. If, as M. Treviranus concludes, the vessels *k*, *k*, *k*, *k*, *fig.* 82, correspond to the biliary vessels of the Insecta *d*, *d*, *figs.* 37 and 38, (and for our own part we cannot but agree with that distinguished physiologist,) then it would appear, as M. Audouin remarks, that the animal referred to is indeed destitute of an intestinal canal properly so called:

"If," says M. Audouin, "the observations of M. Treviranus are correct, and the four vessels which he describes are really analogous to the biliary tubes of insects, we do not hesitate to consider all the part which precedes and is intermediate to them and the four sacs, as the stomach, or chylific cavity. It would thus result, that the *tegenaria domestica* would be deprived of an intestine, properly so called; and would possess no part destined to transmit along a greater or less extent the *residua* of the digestive process. And, indeed, such residua must necessarily be very inconsiderable in an animal which is sustained by juices, and these already animalized. We are, indeed, led to this conclusion by the structure presented by the hemipterous insects, which are nourished, like the spiders, by suction, and which also have the intestines, properly so called, so short that the biliary vessels, which always accompany the posterior extremity of the stomach, are found close to the anus."

The importance of these conclusions, assuming them to be just, in relation to the functions which the liver performs in the animal economy, is manifest; and it is a subject which well merits the attention of the physiologist to enquire into the peculiarities of configuration and structure of this organ and its ducts, as exhibited in the several tribes into which the animal kingdom may naturally be divided.

The account of the circulatory system of the Arachnidans, and the history of the gradual development of the ova, are peculiarly interesting, especially the latter, which admirably exemplifies the patient observation combined with a spirit of enlightened and ingenious research of the author. We should lament to be compelled to confine our researches to this brief notice, were it not that the article itself is readily accessible to all who are desirous of information upon the subject. We trust that the succeeding articles on other classes of the animal kingdom will be written in the same clear and comprehensive manner as those now before us; and there is little doubt but that the whole work will then supply an important chasm in English Medical Literature. It is to be wished, however, that Dr. Todd had embraced within his plan a summary of the peculiarities of vegetable structure and vegetable physiology. That much light is capable of being thrown upon the structure and functions of animals from investigating the organs of plants, is evident from the researches of MM. Dutrochet and Raspail; and the department of Animal Chemistry would not have lost interest by being considered in connexion with and in relation to the analogous products afforded by the vegetable kingdom. In reference to this latter subject, viz. Animal Chemistry, we cannot but observe that the very brief notice of the animal acids by Mr. Brande is meager and unsatisfactory. Some of these are, we find, to be excluded because they have the misfortune to occupy, not a neutral ground between the animal and vegetable kingdoms, but a sort of border territory appertaining to both. Now if the vegetable chemist is to proceed upon the same principle, these hermaphrodites must remain without a local habitation even in the wide extended and comprehensive domain of natural science; and the oxalic, benzoic, acetic, and other acids, find no resting place. Some general views respecting the nature of the acids which are of animal origin, with a reference to their individual properties, might have been given, and a detailed account of those more strictly belonging to the animal kingdom entered into, either under this head or under the chemistry of the animal products more immediately connected with them.

Considering the objects and plan of this work, the knowledge and industry of the Editor, the splendid array of talent combined in its construction, and the elegant manner in which its mechanical part is executed, we cannot but regard it as one of the most important and valuable ever produced in this country. It has its defects, and we have pointed out some of them; and we trust the editor will not refuse, in this early stage of his labours, to profit by any good advice that may be tendered to him by friendly and impartial criticism, whether public or private.

## Figures and Tables

**Fig. 82. f1:**
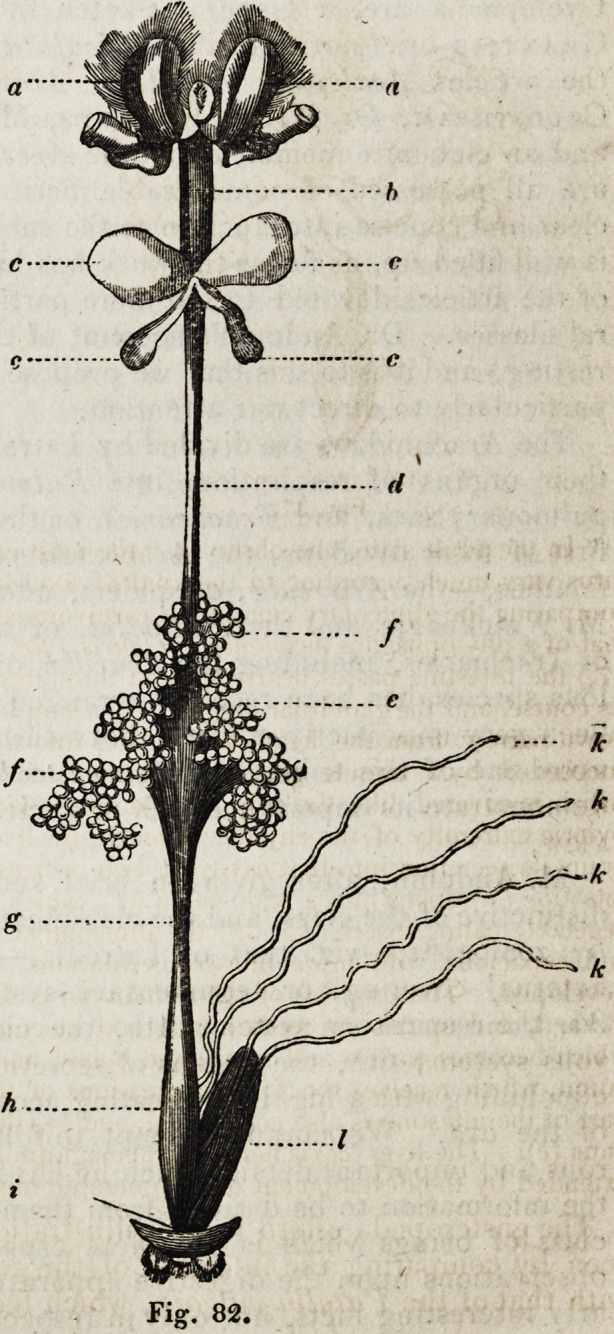


**Fig. 37. f2:**
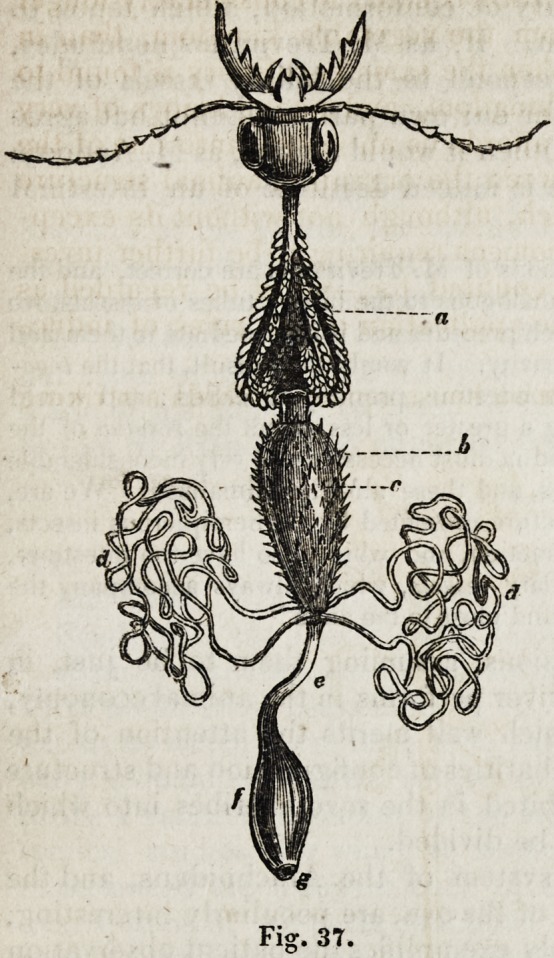


**Fig. 38. f3:**